# Supra-pectoralis major implantation of totally implantable venous access ports in emaciated oncology patients: a single-center retrospective study

**DOI:** 10.3389/fonc.2026.1836109

**Published:** 2026-07-22

**Authors:** Dongdong Yang, Qiuxiang Feng, Liang Xu, Hong Zhu, Xingwei Sun, Jiaming Lu

**Affiliations:** 1 Department of Vascular Surgery, Changshu Hospital Affiliated to Nanjing University of Chinese Medicine, Changshu, Jiangsu, China; 2 Department of Interventional Radiology, The Second Affiliated Hospital of Soochow University, Suzhou, Jiangsu, China

**Keywords:** emaciated patients, oncology, pectoralis major, port-related complications, totally implantable venous access port, venous access

## Abstract

**Background:**

Emaciated oncology patients present unique challenges for totally implantable venous access port (TIVAP) implantation because of limited subcutaneous tissue coverage over the anterior chest wall. Conventional subcutaneous port placement in this population may increase the risk of local complications, including skin erosion, port exposure, and pocket infection. This study aimed to evaluate the feasibility, safety, and short-term clinical outcomes of supra-pectoralis major TIVAP implantation in emaciated oncology patients.

**Methods:**

This retrospective single-center study included 188 consecutive emaciated oncology patients who underwent TIVAP implantation using the supra-pectoralis major technique at the Changshu Hospital Affiliated to Nanjing University of Chinese Medicine between January 2023 and June 2024. Emaciation was defined as a body mass index (BMI) <18.5 kg/m² and subcutaneous fat thickness ≤5 mm at the intended implantation site. All procedures were performed under ultrasound guidance, and catheter tip position was confirmed by fluoroscopy. Technical success, operative time, postoperative complications, and functional port retention during 6–12 months of follow-up were evaluated.

**Results:**

A total of 188 patients were included. The mean age was 70.2 ± 7.8 years, and the mean BMI was 16.3 ± 1.1 kg/m². Technical success was achieved in all patients (100%) with a mean operative time of 24.8 ± 5.8 minutes. During follow-up, device-related complications occurred in 11 patients (5.85%), including port exposure in 2 (1.06%), pocket infection in 4 (2.13%), catheter malposition in 3 (1.60%), and fibrin sheath formation in 2 (1.06%). Four patients (2.13%) required port removal because of complications, while the remaining 184 patients (97.87%) retained a functional port until the end of follow-up or until death from underlying malignancy. During the observation period, 82 patients (43.62%) died from progression of the primary disease.

**Conclusion:**

In this single-center retrospective series, supra-pectoralis major implantation of TIVAPs was technically feasible in emaciated oncology patients and was associated with a high technical success rate and an acceptable short-term complication profile. These preliminary observations suggest that the technique may represent a practical implantation option in this vulnerable population.

## 1 Introduction

Totally implantable venous access ports (TIVAPs) are extensively utilized for long-term central venous access, including the administration of chemotherapy, targeted therapy, immunotherapy, parenteral nutrition, blood products, and repeated blood sampling (1, 2). Compared with external central venous catheters, TIVAPs offer greater convenience, lower maintenance requirements, better patient comfort, and less interference with daily activities. Accordingly, they have become an essential component of supportive care for patients with malignancy.

However, TIVAP implantation may be particularly challenging in certain high-risk populations. One such group comprises emaciated patients, especially those with advanced cancer, cancer cachexia, chronic malnutrition, or substantial treatment-related weight loss. In these patients, the subcutaneous tissue of the anterior chest wall is often markedly thinned, and conventional implantation within the subcutaneous plane may result in insufficient soft-tissue coverage over the port chamber. Consequently, the port may exert excessive pressure on the overlying skin, thereby increasing the risks of pain, delayed wound healing, skin thinning, erosion, wound dehiscence, and eventual port exposure. Once exposure occurs, device removal is often unavoidable, which may interrupt ongoing oncologic therapy and necessitate additional invasive procedures.

Epidemiological studies have shown that malnutrition affects 30% to 80% of patients with malignant tumors (3, 4). In this population, an estimated 30% to 50% of patients experience pronounced emaciation or cancer-associated cachexia, usually defined by a BMI <18.5 kg/m² or involuntary weight loss greater than 10%–15% over a 6-month interval (5–7). This nutritional depletion is particularly common in patients with gastrointestinal malignancies. Data from the INSCOC database indicate that the prevalence of malnutrition in this subgroup may reach 50% to 80% (8, 9). Because anticancer treatment is often prolonged and cyclical, these patients frequently require dependable long-term central venous access (10).

Importantly, cachexia itself has been identified as an independent risk factor for serious postoperative complications. Previous studies have reported that the incidence of port exposure and local ischemic necrosis in emaciated patients ranges from 6.6% to 10.4%, while the rate of pocket infection may reach 7.7% (11, 12). These rates are notably higher than those observed in normal-weight populations, in whom port exposure and infection are generally reported at approximately 0.8%–1.5% and 1.2%–1.8%, respectively (13–15). From a mechanistic perspective, this elevated risk is likely related to severe depletion of the protective subcutaneous fat layer. As the soft-tissue buffer over the chest wall diminishes, the cushioning effect decreases and the mechanical stress generated by the rigid port chamber is transmitted more directly to the overlying skin. In severe cases, local pressure may exceed tissue tolerance, impair capillary perfusion, and lead to microcirculatory compromise, ischemia, and cutaneous necrosis.

In addition to increasing the risk of skin-related complications, local soft-tissue insufficiency may also compromise device stability. In patients with markedly reduced subcutaneous fat, the port chamber may be more prone to prominence, shifting, rotation, or persistent discomfort during repeated puncture. These issues are especially relevant in oncology practice, where ports are often required to remain functional over prolonged periods and to withstand repeated access in routine clinical use. Nevertheless, although the safety of TIVAPs has been extensively studied in general oncology populations, the optimal implantation strategy for emaciated patients has not been adequately defined.

Several alternative approaches have been explored for patients with limited chest wall soft tissue, including smaller port devices and upper extremity ports. However, miniaturized port systems do not fully resolve the problem of inadequate tissue coverage, and their stability may remain suboptimal in severely cachectic patients. Arm ports avoid the anterior chest wall, but they may be associated with upper extremity discomfort, limitations in arm mobility, and a higher risk of upper extremity venous thrombosis in some settings (16–18). Therefore, a practical and anatomically adapted chest port strategy remains clinically desirable.

The pectoralis major region provides a relatively firm and well-vascularized anatomical substrate. We hypothesized that creating the port pocket superficial to the pectoralis major muscle, rather than within a loosely supported superficial subcutaneous plane, might provide better mechanical support and improved local soft-tissue adaptation in emaciated patients. In our center, this supra-pectoralis major implantation strategy was adopted to address the specific challenge of chest wall thinning in patients with limited subcutaneous reserve. Rather than establishing a deep subcutaneous pouch, this technique was designed to construct a secure and well-anchored port foundation superficial to the pectoralis major muscle, while maintaining adequate tissue coverage and ensuring ease of repeated access.

A retrospective review was performed to evaluate the practicality, procedural safety, and short-term results of supra-pectoralis major TIVAP implantation in a consecutive series of emaciated patients from one center. We focused on technical success, procedure-related safety, port-related complications, and functional port retention during follow-up. By addressing a clinically vulnerable and often underrepresented population, this study aimed to provide practical evidence for individualized venous access planning in oncology care.

## 2 Materials and methods

### 2.1 Study design and patient population

This retrospective single-center study evaluated emaciated oncology patients who underwent TIVAP implantation using a supra-pectoralis major implantation technique at the Changshu Hospital Affiliated to Nanjing University of Chinese Medicine, between January 2023 and June 2024. Consecutive eligible patients were screened during the study period, and a total of 188 patients who met the inclusion criteria were enrolled in the final analysis.

The study protocol was approved by the Medical Ethics Committee of the Changshu Hospital Affiliated to Nanjing University of Chinese Medicine. The study was conducted in accordance with the Declaration of Helsinki. Because of the retrospective nature of the study, the requirement for written informed consent was waived by the institutional review board. All patient data were anonymized before analysis.

A total of 188 patients were included. The cohort comprised 144 men and 44 women, with a mean age of 70.2 ± 7.8 years (range, 45–87 years). Gastrointestinal malignancies accounted for the majority of cases (n = 157), followed by lung cancer (n = 11), hematological malignancies (n = 5), and other solid tumors (n = 15).

Patients were eligible if they required long-term central venous access for chemotherapy, targeted therapy, immunotherapy, parenteral nutrition, or other oncologic supportive treatment; had emaciation defined as a BMI <18.5 kg/m² according to World Health Organization criteria; and had a preoperative ultrasonographic measurement of subcutaneous tissue thickness ≤5 mm at the intended anterior chest wall implantation site. This threshold was selected because limited soft-tissue coverage is generally considered a risk factor for port-related skin complications in emaciated patients.

Exclusion criteria were prior radiotherapy to the ipsilateral chest wall, severe chronic obstructive pulmonary disease or advanced emphysema, previous surgery involving the ipsilateral pectoralis major region or overlying fascia, autoimmune connective tissue disease, and long-term systemic glucocorticoid use (>10 mg/day prednisone equivalent). These conditions were excluded because they may impair wound healing, compromise local tissue integrity, or affect the safety and stability of port implantation.

### 2.2 Preoperative optimization and surgical protocol

All patients underwent routine preoperative evaluation, including complete blood count, coagulation testing, and biochemical assessment of renal and hepatic function. High-frequency ultrasonography with a linear transducer (≥12 MHz) was used to assess the planned implantation site, including subcutaneous tissue thickness and the integrity of the superficial pectoralis major fascia. In selected patients, non-contrast chest computed tomography with thin-slice reconstruction was also performed to assess thoracic contour and exclude marked chest wall deformities that might interfere with appropriate port seating.

Whenever feasible, preoperative nutritional and metabolic optimization was undertaken. Recommended target parameters included serum albumin ≥35 g/L and hemoglobin >100 g/L. These values were pursued as optimization goals rather than strict prerequisites, and implantation was not delayed in patients who could not achieve these targets within a clinically acceptable interval. Before implantation, skin preparation was performed using a 2% chlorhexidine gluconate cleansing protocol for three consecutive days (19). Low-profile port systems were preferentially selected to reduce pressure on the overlying skin.

**Figure 1:**
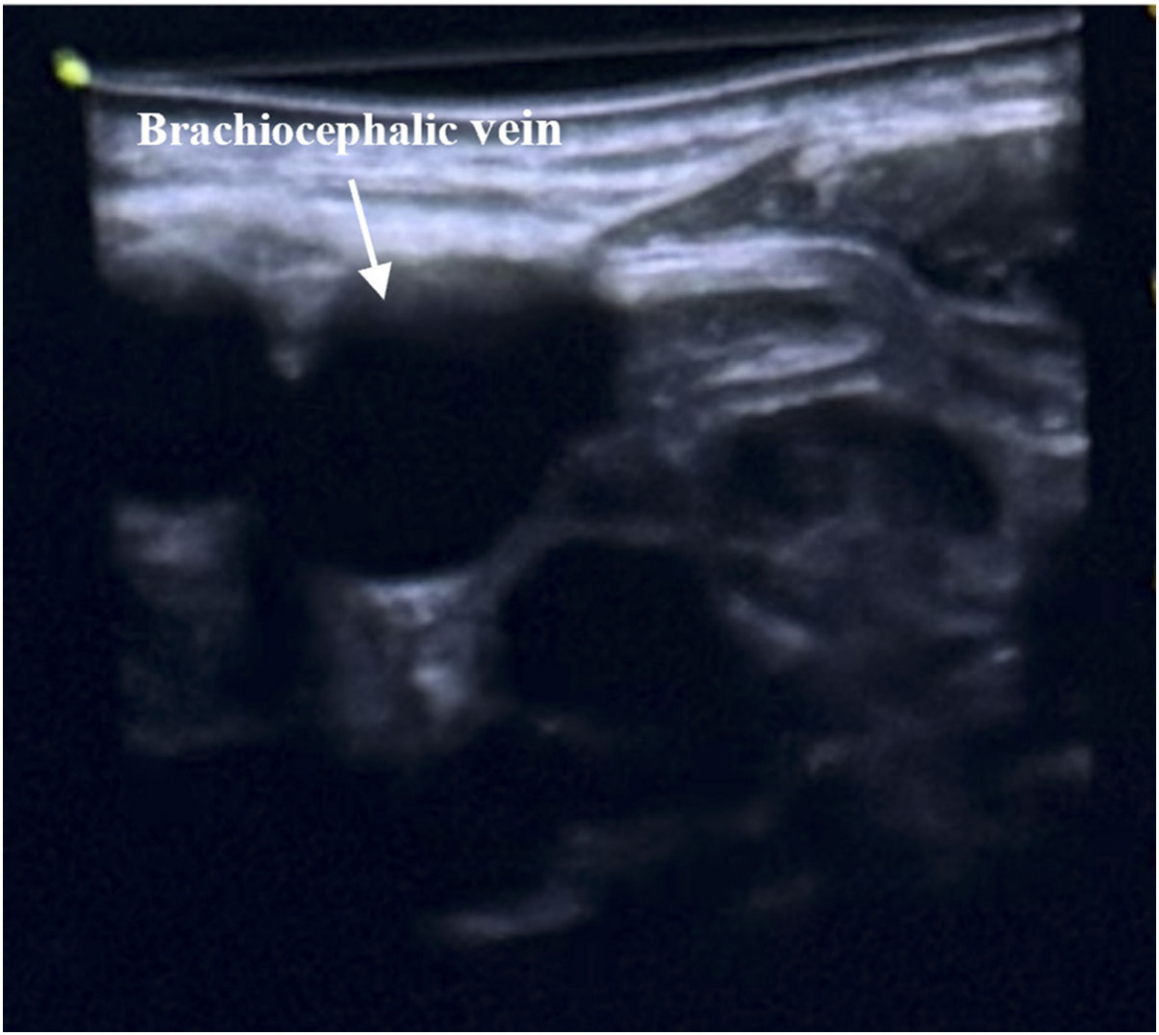
Ultrasound-guided venous cannulation.

The procedure was performed with the patient in the supine position, the head turned contralaterally, and the ipsilateral shoulder slightly lifted with a radiolucent cushion. After local anesthetic infiltration using 1% lidocaine, venous cannulation was preferentially performed through the right internal jugular vein under ultrasound guidance. The brachiocephalic vein or subclavian vein was used when necessary (**Figure 1**). A guidewire was advanced into the superior vena cava under fluoroscopic guidance, followed by tract dilation and catheter insertion through a peel-away sheath. Correct intravascular placement was confirmed by blood aspiration and saline flushing.

**Figure 2:**
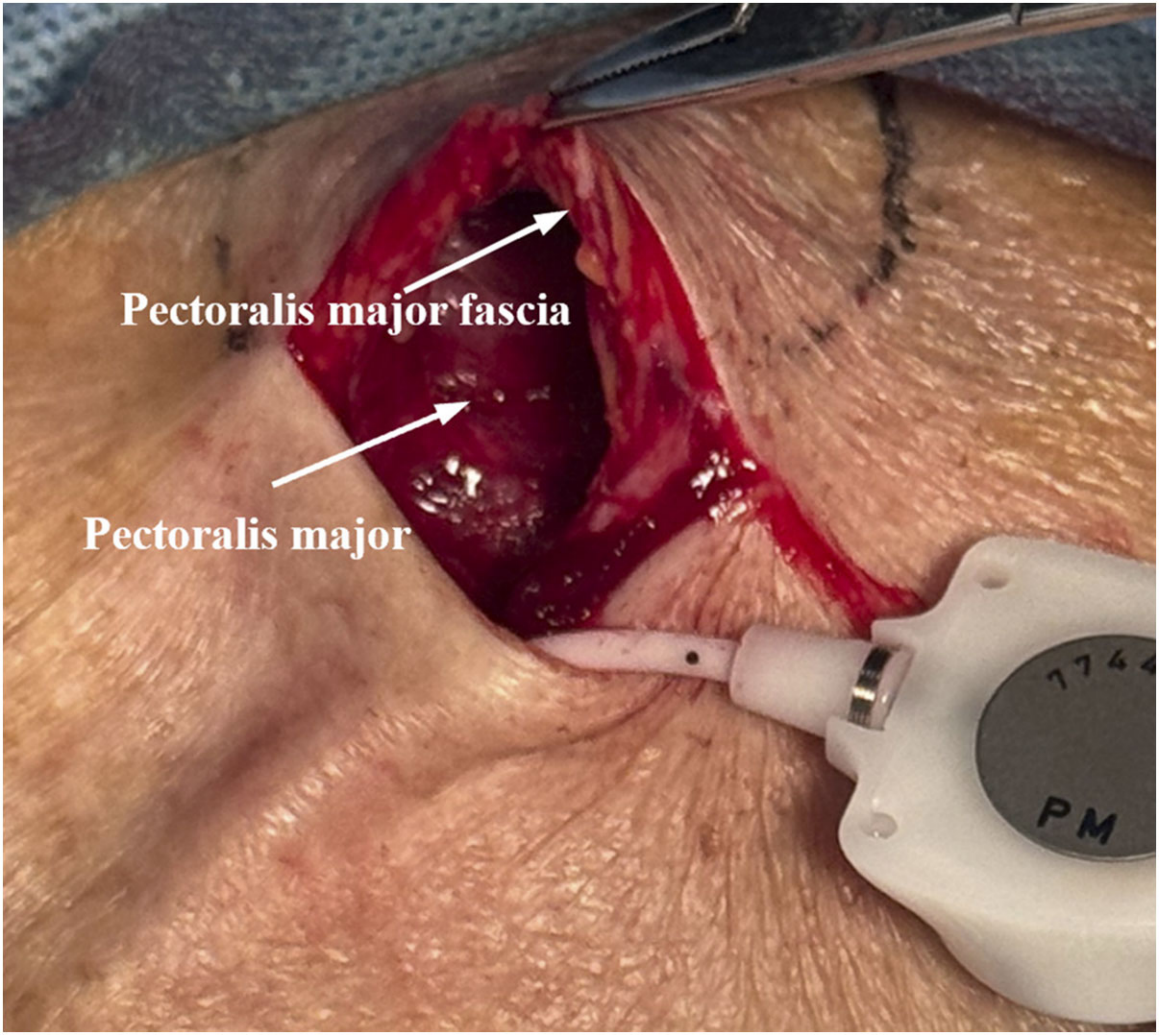
Creation of the supra-pectoralis major port pocket.

Attention was then directed to creation of the port pocket. A transverse chest wall incision of approximately 2.5 cm was made, and blunt dissection was carried down to the superficial fascia of the pectoralis major muscle. The fascia was incised at the level of the incision, and a pocket matched to the dimensions of the port base was created directly on the surface of the pectoralis major muscle beneath the fascial plane (**Figure 2**). Dissection was performed carefully to minimize trauma to the muscle and local vessels, and meticulous hemostasis was maintained throughout the procedure.

**Figure 3:**
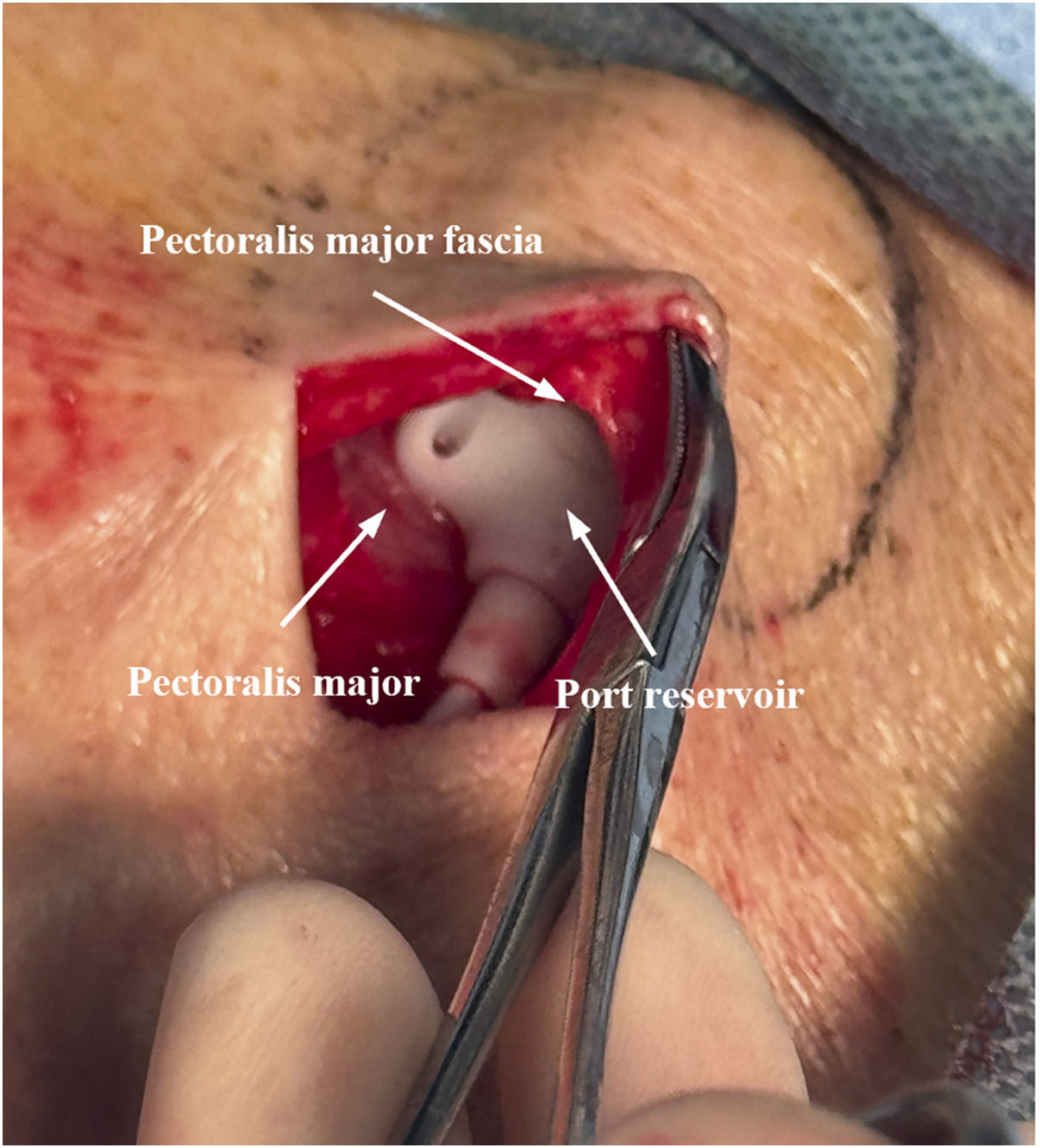
Port placement.

**Figure 4:**
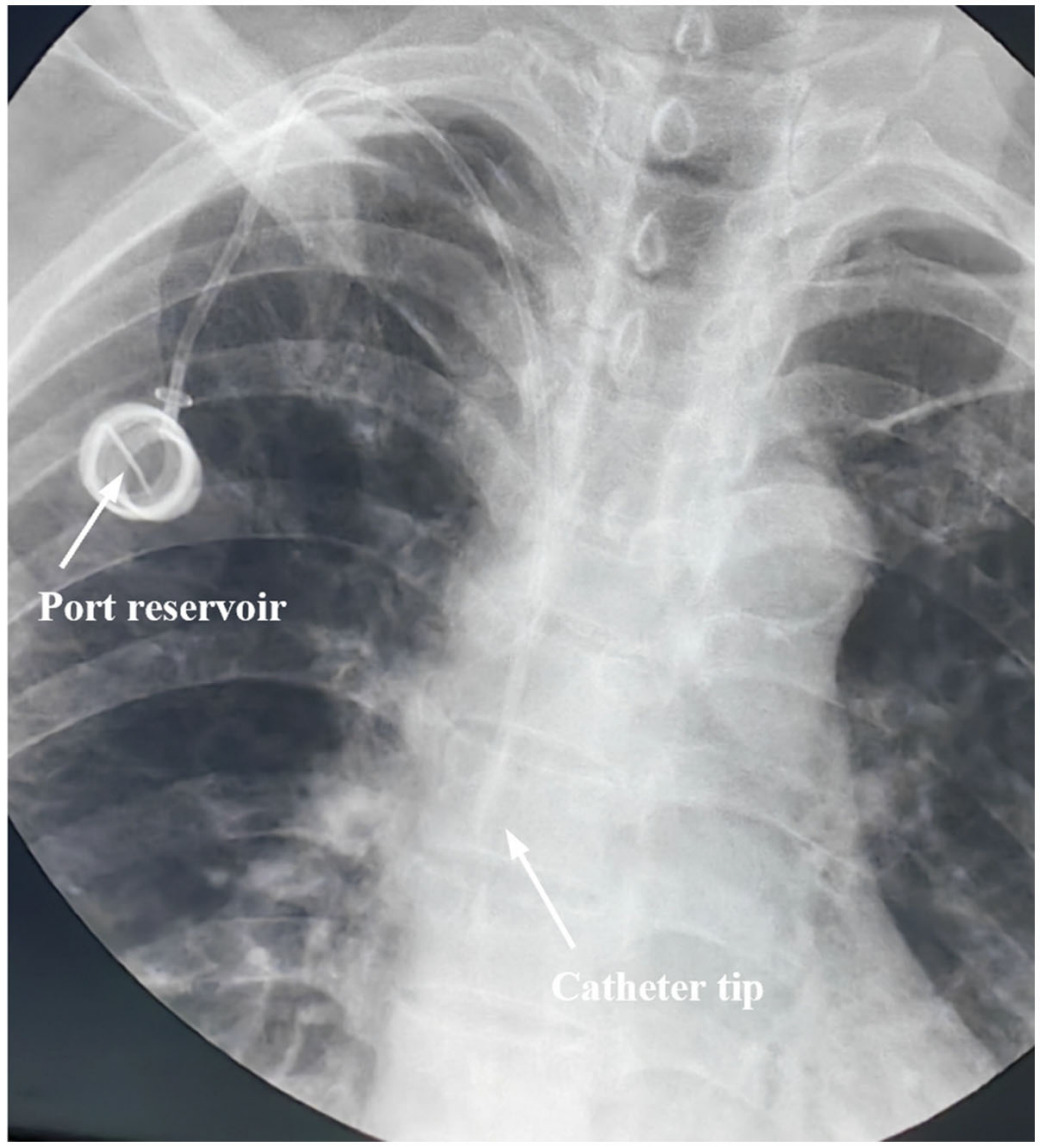
Final fluoroscopic confirmation.

Subcutaneous tunneling was performed between the neck puncture site and the chest wall reservoir pocket. Under fluoroscopy, the catheter was introduced through the tunnel, shortened as needed, and finally positioned at the cavoatrial junction. The port chamber was then placed within the supra-pectoralis pocket directly over the pectoralis major muscle (**Figure 3**). If intraoperative assessment suggested insufficient stability, additional fixation sutures were placed as needed. The pectoral fascia and the overlying skin and subcutaneous tissue were closed as a composite layer using interrupted non-absorbable sutures. Port patency was confirmed using a non-coring Huber needle, followed by saline flushing and sterile dressing application. Final fluoroscopic imaging was obtained to confirm catheter position and overall device configuration (**Figure 4**).

### 2.3 Postoperative management and study endpoints

After implantation, a moderate local compression dressing was routinely applied for 24–48 hours. Early postoperative ultrasonography was performed at 24 hours to assess for peri-port fluid collection. If a clinically significant seroma was identified, ultrasound-guided aspiration was performed under sterile conditions when indicated. A follow-up ultrasound examination was also performed on postoperative day 7 to evaluate local healing and port pocket adaptation. Additional compression support was applied when needed according to the local condition of the wound and device bed.

During treatment intervals, ports were maintained according to institutional protocols, including regular flushing with heparinized saline. After implantation, patients were followed for 6–12 months, with scheduled assessments at 1, 3, 6 and 12 months. Follow-up surveillance was conducted through routine inpatient and outpatient clinical evaluations, supplemented by review of electronic medical records, and focused on device-related complications, including port exposure, pocket infection, wound-related complications, catheter dysfunction, catheter-related thrombosis, and the need for port removal or revision. For patients who died during follow-up, all available medical records before death were reviewed, and, when necessary, family members were contacted by telephone to identify any documented device-related events.

The primary study endpoints were: (1) technical success, defined as successful port implantation with satisfactory catheter tip position; (2) port exposure, defined as visible exposure of the port chamber or exposure secondary to wound dehiscence; and (3) pocket infection, defined by local inflammatory signs with or without purulent discharge requiring clinical management. Secondary endpoints included operative time, catheter-related complications such as fibrin sheath formation, malposition, catheter fracture, and pinch-off syndrome, as well as functional port retention during follow-up.

### 2.4 Statistical analysis

SPSS version 26.0 (IBM Corp., Armonk, NY, USA) was used for data processing. Normally distributed continuous variables are given as mean ± standard deviation, and categorical variables are reported as counts with corresponding percentages. Given the retrospective observational design of the study, analyses were primarily descriptive. The observed clinical outcomes were interpreted in the context of previously published data on conventional TIVAP implantation techniques.

## 3 Results

### 3.1 Baseline characteristics

A total of 188 emaciated oncology patients were included in this study. The mean age was 70.2 ± 7.8 years, and 144 patients were male and 44 were female. The mean body mass index was 16.3 ± 1.1 kg/m². The study period ranged from January 2023 to June 2024. During follow-up, 82 patients (82/188, 43.62%) died because of progression of the underlying malignancy or related clinical deterioration, with a median follow-up duration of 8 months (range, 6–11 months) in these deceased patients.

The most common primary malignancies were gastrointestinal tumors (157/188, 83.51%), followed by lung cancer (11/188, 5.85%), hematological malignancies (5/188, 2.66%), and other solid tumors (15/188, 7.98%). TIVAP implantation was performed mainly for chemotherapy (152 patients), followed by parenteral nutrition/supportive care (29 patients), targeted therapy (5 patients), and immunotherapy (2 patients). The internal jugular vein was the most frequently used access route (162 patients), followed by the brachiocephalic vein (21 patients) and the subclavian vein (5 patients) (**Table 1**).

**Table 1:** Baseline characteristics of the study population (n = 188).

Variable	Value
Age, years	70.2 ± 7.8
Sex, male/female	144/44
BMI, kg/m²	16.3 ± 1.1
Study period	January 2023 to June 2024
Follow-up duration, months	6–12
Primary malignancy	
– Gastrointestinal malignancies	157 (83.51%)
– Lung cancer	11 (5.85%)
– Hematological malignancies	5 (2.66%)
– Other solid tumors	15 (7.98%)
Indication for TIVAP implantation	
– Chemotherapy	152
– Targeted therapy	5
– Immunotherapy	2
– Parenteral nutrition/supportive care	29
Venous access route	
– Internal jugular vein	162
– Brachiocephalic vein	21
– Subclavian vein	5

Continuous variables are presented as mean ± standard deviation, and categorical variables as n (%).

### 3.2 Procedural outcomes

All 188 patients successfully underwent TIVAP implantation using the supra-pectoralis major approach, resulting in a technical success rate of 100%. The mean operative time was 24.8 ± 5.8 minutes. No intraoperative complications were observed. All ports were successfully used for the intended treatment within 24–48 hours after implantation, which was consistent with routine use following the conventional technique.

### 3.3 Postoperative complications

During the 6- to 12-month follow-up period, postoperative complications occurred in 11 of 188 patients (5.85%). These included port exposure in 2 patients (1.06%), pocket infection in 4 (2.13%), catheter malposition in 3 (1.60%), and fibrin sheath formation in 2 (1.06%).

Port exposure occurred in 2 patients and presented as progressive thinning and ulceration of the skin overlying the port, followed by local tissue breakdown and exposure of the device. Both patients underwent local debridement; however, because the sterile barrier had been compromised, port removal was ultimately required.

Pocket infection was identified in 4 patients. Clinical manifestations included local erythema, increased skin temperature, tenderness or resting pain, and increasing visibility of the port contour beneath the skin. Management included systemic antibiotic therapy, ultrasound-guided pocket irrigation when indicated, local antimicrobial dressings, and nutritional support. In 2 patients, these measures resulted in resolution of infection and preservation of the port, whereas the other 2 required device removal because of persistent infection.

Catheter malposition occurred in 3 patients and was manifested by reduced infusion flow, increased resistance during injection, or difficulty with blood aspiration. Imaging confirmed catheter tip migration in all cases. Endovascular repositioning under fluoroscopic guidance was successfully performed in all 3 patients, with restoration of normal catheter function.

Fibrin sheath formation was observed in 2 patients who presented with reduced infusion flow and impaired blood aspiration. Ultrasonography confirmed the diagnosis, and both patients were successfully treated with intraluminal urokinase locking, resulting in restoration of catheter patency.

No cases of pneumothorax, hemothorax, catheter fracture, port inversion, catheter-port disconnection, or pinch-off syndrome were observed during follow-up. Overall, 4 patients (2.13%) required port removal because of severe complications, specifically port exposure or infection unresponsive to conservative treatment. At the end of follow-up, 184 patients (97.87%) retained functional ports (**Table 2**).

**Table 2:** Procedural outcomes and complications, management, and clinical outcomes.

Variable	Value
Technical success	188/188 (100%)
Operative time, min	24.8 ± 5.8
Intraoperative complications	0
Overall postoperative complications	11/188 (5.85%)
– Port exposure	2/188 (1.06%)
– Pocket infection	4/188 (2.13%)
– Catheter malposition	3/188 (1.60%)
– Fibrin sheath formation	2/188 (1.06%)
Management of port exposure	Debridement + port removal in 2 patients
Management of pocket infection	Conservative treatment successful in 2 patients; port removal in 2 patients
Management of catheter malposition	Endovascular repositioning successful in 3 patients
Management of fibrin sheath formation	Urokinase locking successful in 2 patients
Pneumothorax	0
Hemothorax	0
Catheter fracture	0
Port inversion	0
Catheter-port disconnection	0
Pinch-off syndrome	0
Port removal due to complications	4/188 (2.13%)
Functional port retention at end of follow-up	184/188 (97.87%)

Values are presented as n (%) unless otherwise indicated.

## 4 Discussion

### 4.1 Technical advantages and mechanistic analysis

In this retrospective single-center cohort of emaciated oncology patients, supra-pectoralis major TIVAP implantation was technically feasible in all 188 cases, with a technical success rate of 100%. During 6–12 months of follow-up, port exposure (1.06%) and pocket infection (2.13%) were both uncommon, and 97.87% of patients retained a functional port until the end of follow-up or until death from underlying disease. Although these numerical outcomes appear favorable in a population traditionally considered high-risk, several caveats limit the strength of these observations. First, 82 patients (43.62%) died during the observation period from progression of the primary malignancy. Substantial competing mortality of this magnitude reduces the effective time at risk for late device-related events and may therefore have led to underestimation of delayed complications, such as chronic skin thinning, indolent pocket infection, or late catheter-related thrombosis. Second, the study was uncontrolled, and direct comparisons with historical rates reported for conventional subcutaneous implantation are inevitably confounded by heterogeneity in patient selection, complication definitions, perioperative management, and follow-up length. Therefore, the present findings should be interpreted as preliminary evidence of feasibility and short-term safety, rather than as demonstration of superiority over conventional techniques.

The mechanistic explanations offered below are speculative and are presented only to provide a biologically plausible framework for the observed clinical results; none of them was directly measured in the present study. From an anatomical standpoint, we hypothesize that supra-pectoralis major placement may improve the mechanical interaction between the rigid port chamber and the surrounding soft tissues. In emaciated patients, conventional subcutaneous implantation situates the chamber beneath a markedly thinned skin–fat envelope, in which soft-tissue buffering may be insufficient to dissipate the focal pressure exerted by the port base. Repeated puncture, respiratory motion, postural change, and daily friction from clothing may further amplify this local mechanical load, and, over time, contribute to skin thinning, impaired microcirculation, delayed healing, and eventual exposure. By seating the port on the surface of the pectoralis major beneath the pectoral fascia, this technique may redistribute mechanical load over a broader and more structurally coherent plane, potentially reducing peak dermal stress. This proposed mechanism, however, remains hypothetical and would require dedicated biomechanical or imaging-based evaluation to confirm.

A second potential contribution of this approach may lie in improved chamber stability. In cachectic patients, atrophic subcutaneous tissue is loose and offers limited resistance to repetitive shear forces, so that the implanted chamber may be more prone to micromotion, tilting, or displacement during coughing, deep respiration, or upper-limb movement. We speculate that the pectoralis major, together with its overlying fascia, provides a firmer structural foundation that may better resist multidirectional traction and allow the device to move more synchronously with the chest wall. If confirmed, such improved anchoring could plausibly reduce the risk of port malrotation, misaligned puncture, catheter kinking, and tip migration. While our data cannot directly test this hypothesis, the absence of catheter fracture, port inversion, pinch-off syndrome, or catheter–port disconnection in the present cohort is at least consistent with adequate mechanical stability under routine clinical use.

A third proposed advantage relates to local tissue biology. The pectoralis major region is relatively well vascularized compared with the severely thinned superficial tissue envelope observed in cachectic patients, and adequate perfusion is essential for wound healing, immune surveillance, and resistance to pressure-related injury. In patients with advanced malignancy—many of whom also present with hypoalbuminemia, anemia, chemotherapy exposure, and immunosuppression—situating the device on a more vascularized and structurally coherent bed may plausibly favor pocket healing. In addition, closure of the pectoral fascia together with the overlying skin and subcutaneous tissue as a composite layer provides an additional soft-tissue barrier between the chamber and the skin surface, which we hypothesize may reduce the risk of superficial erosion and secondary bacterial contamination. As with the mechanical hypotheses above, these interpretations remain inferential and should be regarded as hypotheses requiring further validation in prospective, mechanistically oriented studies.

Taken together, the observed short-term outcomes are compatible with, but do not prove, the anatomical and biological rationale underlying this technique. The favorable complication profile in this cohort likely reflects the combined effect of a modified pocket plane, ultrasound-guided venous access, fluoroscopically confirmed catheter tip placement, and structured postoperative surveillance, rather than any single factor in isolation. Accordingly, we consider the supra-pectoralis major approach a mechanistically plausible and clinically feasible option in emaciated oncology patients, whose comparative effectiveness against conventional subcutaneous placement remains to be established.

### 4.2 Multimodal optimization strategies and clinical recommendations

The favorable short-term outcomes observed in this cohort were most likely attributable not to the modified pocket plane alone, but to the combined effect of careful patient selection, anatomically tailored pocket construction, standardized venous access, and structured perioperative surveillance. The following considerations are therefore presented as practice-oriented implications derived from the present experience, rather than as independently validated determinants of outcome.

Preoperative planning should be based on individual morphometry and local chest wall anatomy. In patients with a BMI <18.5 kg/m² and subcutaneous adipose thickness <5 mm, the supra-pectoralis major approach may represent a practical alternative to conventional subcutaneous implantation, in which the thin superficial envelope often provides insufficient cushioning and predisposes to pressure-related skin compromise. In cases of extreme cachexia with subcutaneous fat ≤3 mm, adjunctive use of an “artificial biological buffer” such as an absorbable collagen membrane or acellular dermal matrix may be considered, as prior biomechanical work suggests that interposition of a collagenous matrix may reduce peak dermal pressure and mitigate compressive microcirculatory ischemia (20, 21). Because this adjunct was not systematically evaluated in the present study, it should be regarded as a selective option for highly vulnerable patients. Preoperative ultrasound should extend beyond venous mapping to include measurement of local tissue thickness and identification of relatively preserved soft-tissue areas for pocket siting.

Chamber positioning and vascular access should be considered together. The geometric center of the port base should be at least 2 cm from rigid osseous landmarks such as the clavicle or sternocostal junctions in order to limit focal stress concentration in patients lacking an adipose cushion. For venous access, the right internal jugular vein (RIJV) remains the preferred route when its luminal diameter is ≥8 mm, given its relatively straight course into the superior vena cava; when the RIJV is unsuitable, the brachiocephalic vein (BCV) is an acceptable alternative because of its large caliber and favorable convergence angle into the central venous circulation (22). Regardless of entry site, the objective is a linear and atraumatic catheter course that minimizes malposition, thrombosis, and secondary dysfunction.

Preoperative nutritional and hematologic optimization may influence wound healing and infectious risk. In the present practice, serum albumin ≥35 g/L and hemoglobin >100 g/L were considered desirable targets, given the association of hypoalbuminemia and anemia with impaired repair. These thresholds are frequently unattainable in patients with cancer cachexia or treatment-related marrow suppression, and implantation should not be routinely deferred when reliable central venous access is clinically required. When targets could not be met, short-term nutritional support, selective correction of anemia, and closer wound surveillance were used as appropriate. Such optimization complements, rather than replaces, meticulous surgical technique.

Intraoperatively, the pocket was tailored to the diameter of the port base with an approximately 5-mm margin, producing a close-fitting but non-compressive configuration intended to limit dead space, seroma formation, and chamber micromotion without imposing undue local tension. Before wound closure, an intraoperative cough test or simulated Valsalva maneuver was used to assess dynamic stability; visible movement greater than 1 mm prompted additional multi-point anchoring sutures, placed carefully to preserve pectoralis major microvasculature. The pectoral fascia was not closed as a separate layer but was incorporated with the overlying skin and subcutaneous tissue as a composite layer, using interrupted non-absorbable sutures removed 10–12 days postoperatively. This closure strategy was intended to minimize buried foreign material and associated inflammatory stimulation in patients with limited healing reserve. The catheter tip was positioned at the cavoatrial junction (typically T7–T8), where the high-flow hemodynamic environment facilitates infusate dilution and may reduce fibrin deposition; this hemodynamic “washout effect,” associated with shear stress >15 dyn/cm², is thought to lessen endothelial irritation, chemical phlebitis, and thrombosis (23–25). Optimized pocket construction should therefore be integrated with reliable fluoroscopic tip confirmation.

Postoperative care emphasized early stabilization and vigilant local surveillance. Within the first 48 hours, moderate elastic compressive dressings may reduce residual dead space and improve chamber–tissue adherence; peri-port seromas >5 mm on ultrasound may be considered for early sterile aspiration. In emaciated patients, subtle erythema, pressure marks, or blanching may precede overt erosion, and prompt recognition may allow intervention before progression to exposure or pocket infection. During maintenance, transparent semipermeable dressings should be replaced every 5–7 days; in patients with cutaneous hypersensitivity or localized epidermal injury, hydrocolloid dressings may be considered when clinically indicated (26). For continuous infusions lasting more than 4 hours, chlorhexidine-impregnated dressings are recommended around the puncture site (27), as prolonged infusion sustains an interface favoring bacterial colonization; such dressings provide persistent antimicrobial protection against common cutaneous organisms and may reduce local and catheter-related infection (28). Thromboprophylaxis and catheter surveillance should be individualized; systemic anticoagulation may be guided by the Caprini Thrombosis Risk Assessment score, and locoregional thrombolysis may be considered when color Doppler ultrasonography demonstrates fibrin sheath coverage exceeding 30% of the catheter surface, in order to prevent complete occlusion and preserve long-term access (29). In routine use, positive-pressure locking, immediate flushing after blood aspiration or high-viscosity infusions (blood products, total parenteral nutrition, or lipid emulsions), and an increased flushing volume (e.g., 20 mL) help maintain luminal patency (24).

Taken together, the present findings indicate that the short-term performance of the supra-pectoralis major technique is best understood within a broader perioperative framework rather than as a consequence of pocket depth modification alone. Emaciated oncology patients typically combine fragile skin, limited nutritional reserve, impaired healing capacity, prolonged treatment exposure, and repeated port access; in this setting, outcome optimization depends on coordinated attention to preoperative assessment, anatomically appropriate implantation, and structured postoperative care. The supra-pectoralis major technique should thus be regarded as one component of an individualized venous access strategy for patients with severely compromised chest wall soft tissue.

### 4.3 Study limitations

The present study has several limitations that should be taken into consideration. First, as a single-center retrospective analysis based on historical medical records, the study is inherently susceptible to selection bias, information bias, and unmeasured confounding. The absence of a contemporaneous control group undergoing conventional implantation within the same study framework limits the strength of causal inference. As a result, although the observed complication profile appears favorable, comparisons with previously published rates should be interpreted cautiously.

Second, although the current sample size is clinically meaningful for this relatively specific patient population, it remains insufficient for detailed subgroup analysis. It would be valuable to determine whether outcomes differ according to the severity of cachexia, primary tumor type, nutritional indicators, prior treatment exposure, or venous access route. Larger multicenter studies will be necessary to explore these issues and to better define the patient subgroups most likely to benefit from this technique.

Third, the relatively short follow-up duration of 6 to 12 months may be insufficient to fully support conclusions regarding the long-term safety and durability of supra-pectoralis major TIVAP implantation. This limitation is closely related to the survival characteristics of the study population, as emaciation and cachexia commonly occur in patients with advanced malignancy and are themselves associated with shortened overall survival. Consequently, extended follow-up is often difficult to obtain because of disease progression or death within a relatively short interval after implantation. The present observations therefore primarily reflect short-term clinical performance, and late complications such as delayed skin erosion, chronic infection, catheter-related thrombosis, or catheter dysfunction may not have been fully captured. Prospective studies with longer follow-up are warranted to further clarify the long-term outcomes of this technique.

Fourth, the mechanistic interpretations proposed in this discussion are based primarily on clinical observation and anatomical reasoning rather than direct biomechanical measurement. Although it is plausible that improved stress distribution, enhanced chamber stability, and better local tissue support contribute to the favorable outcomes observed, these hypotheses were not formally tested. Future studies incorporating imaging-based motion analysis, pressure-distribution assessment, and quantitative evaluation of soft-tissue thickness may help clarify the biological and mechanical basis of this technique.

Finally, this study focused primarily on feasibility and complication outcomes, whereas other clinically relevant endpoints were not systematically assessed. Patient-reported outcomes such as pain during puncture, cosmetic satisfaction, sense of foreign-body awareness, impact on daily activity, nursing convenience during port access, and treatment interruption related to device events may provide a more comprehensive understanding of the clinical utility of this approach. Future prospective, randomized controlled trials are warranted to validate the long-term efficacy of this technique and to establish robust, evidence-based recommendations for TIVAP implantation in emaciated patients.

## 5 Conclusion

In this retrospective single-center study, supra-pectoralis major TIVAP implantation was technically feasible in emaciated cancer patients and was associated with a high short-term functional port retention rate and an acceptable incidence of port exposure and pocket infection. These preliminary findings indicate that modification of the implantation plane may represent a practical strategy for patients with limited anterior chest wall soft-tissue coverage. From an anatomical perspective, the potential clinical value of this approach may be related to enhanced local support of the port chamber and improved adaptation to the emaciated chest wall, although these mechanisms were not formally tested in the present study. When combined with careful patient selection, appropriate pocket positioning, and standardized perioperative management, this technique appears to be a reasonable option for individualized venous access planning in this vulnerable population. Given the retrospective, uncontrolled, single-center design and the limited follow-up duration, the present observations should be regarded as hypothesis-generating. Larger prospective multicenter comparative studies would help further clarify its clinical role and comparative performance in venous access management for emaciated oncology patients.

## Data Availability

The raw data supporting the conclusions of this article will be made available by the authors, without undue reservation.
